# Optimization and characterization of zinc oxide nanoparticles synthesized with the assistance of *Bacillus tequilensis* supernatant

**DOI:** 10.1016/j.biotno.2026.03.002

**Published:** 2026-04-06

**Authors:** Elayappan Sindhu, Arumugam Karthikeyan

**Affiliations:** Forest Protection Division, ICFRE-Institute of Forest Genetics and Tree Breeding, Coimbatore, Tamil Nadu, India

**Keywords:** Green synthesis, Zinc oxide nanoparticles, Characterization, Optimization, *Bacillus tequilensis*

## Abstract

Biogenic synthesis of metal oxide nanoparticles offers a novel and environmentally friendly alternative to conventional synthesis methods. The current study utilized the cell-free supernatant of *Bacillus tequilensis* to assist the biosynthesis of Zinc Oxide (ZnO) nanoparticles by employing zinc sulphate monohydrate as precursor. The conditions of the synthesis procedure, such as precursor concentration, pH, and temperature of the reaction mixture, were optimized to improve the nanoparticle yield, stability, and reproducibility. The formation of ZnO nanoparticles was assessed using UV-Vis spectroscopy. Fourier-transform infrared (FTIR) analysis demonstrated the presence of functional groups responsible for the reduction and stabilization of the nanoparticles. The crystalline wurtzite structure of ZnO was established using X-ray Diffraction (XRD). Field-emission scanning electron microscopy (FESEM) with energy dispersive X-ray spectroscopy established the shape of the particles and elemental composition corresponding to Zn and O. Zeta potential analysis indicated the moderate colloidal stability of the synthesized nanoparticles. The findings of the present study demonstrate the feasibility of using *Bacillus tequilensis* as a novel biological system for the controlled synthesis of ZnO nanoparticles using the cell-free supernatant method.

## Highlights

• ZnO nanoparticles synthesized using cell-free supernatant of *Bacillus tequilensis.*
• Optimization of precursor concentration, pH, and temperature.
• Characterization using UV-Vis, FTIR, Zeta potential, DLS, FESEM-EDS, and XRD.
• *Bacillus tequilensis* as novel biogenic agent for assisted ZnO nanoparticle synthesis.

## 1 Introduction

Nanotechnology has enabled the development of metal oxide nanoparticles with diverse applications across biomedical, environmental and agricultural areas, with zinc oxide (ZnO) nanoparticles getting selective attention due to their antimicrobial, antioxidant, anti-inflammatory and anticancer properties, decreased toxicity and improved biocompatibility.^1^^,^^2^

ZnO nanoparticles are conventionally synthesized using physical and chemical methods. However, physical methods often require complicated equipment, skilled manpower and high energy input, while chemical synthesis frequently involves toxic reducing and stabilizing agents that generate environmentally hazardous byproducts.^3^, ^4^, ^5^, ^6^ As a consequence to these factors, green or biological synthesis techniques have emerged as a sustainable alternative, offering advantages including reduced toxicity, energy efficiency, and environmental compatibility.^2^^,^^5^, ^6^, ^7^

Among various biogenic synthesis techniques, the microbe-mediated method is a well-documented, eco-friendly, scalable and feasible approach.^8^ Microorganisms produce a wide range of bioactive compounds, including metabolites and enzymes, which assist in the reduction and stabilization of metal ions into nanoparticles through intracellular or extracellular mechanisms.^9^, ^10^, ^11^, ^12^ Bacterial systems are particularly preferred owing to their ease of cultivation, rapid growth and efficient metal tolerance.^13^^,^^14^ Within bacterial systems, the *Bacillus* species are widely recognised for their role in the green synthesis of nanoparticles due to their ability to reduce biomolecules.^15^ They have been reported to produce various bio-surfactants,especially lipopeptides, which facilitate nanoparticles synthesis.^16^ Hence, *Bacillus* can be considered as a potential agent for a biological nano-factory.^17^

Numerous studies have established the prospective role of *Bacillus subtilis.*^2^, ^18^, ^19^ *B. licheniformis*^18^ and *B. paramycoides*^19^ in synthesizing ZnO nanoparticles. Although the optimization of synthesis parameters has been established for certain *Bacillus* species, these findings are innately species-specific and cannot be directly extrapolated to less-explored *Bacillus* strains with distinct metabolic and extracellular biomolecule profiles. Accordingly, the biogenic potential of several *Bacillus* species remains inadequately investigated in the context of the controlled and reproducible synthesis of ZnO nanoparticles.

Based on previous reports, *Bacillus tequilensis* is known to secrete a range of extracellular biomolecules, including polysaccharides, lipoprotein biosurfactants, and proteins, which include functional groups capable of interacting with metal ions. Earlier studies have established that these biomolecules also exhibit physical and chemical stability under variable temperature and pH conditions, suggesting their applicability for the biogenic synthesis process.^20^ Reports have also suggested that bacterial biosurfactants contribute to the reduction, capping, and stabilization of metal oxide nanoparticles. 21, 22, 23

In addition, *Bacillus tequilensis* belongs to the *Bacillus subtilis* species complex^24^^,^^25^ and has been reported to interact with zinc through solubilization and transformation machinery,^26^ indicating its affinity for zinc-based systems.

Despite these advantageous biochemical characteristics, *Bacillus tequilensis* has not been thoroughly investigated for ZnO nanoparticle synthesis using the cell-free supernatant approach.

Therefore, the present study focuses on the synthesis of ZnO nanoparticles using the cell-free supernatant of *B. tequilensis*, followed by the optimization of synthesis parameters, namely precursor concentration, pH and temperature of the reaction mixture, using UV-Vis spectroscopy. Characterization was carried out using FTIR, XRD, Zeta potential analysis, and FESEM-EDS to determine their physical and chemical features. By presenting *B. tequilensis* as a novel biogenic system for assisting ZnO nanoparticle production and establishing of controlled synthesis conditions, this study advances *Bacillus*-mediated ZnO nanoparticle research beyond commonly explored species.

To clearly position the present study within the context of existing *Bacillus*-mediated ZnO nanoparticle research, a summary of previously reported *Bacillus* species used for ZnO nanoparticle synthesis is provided in Table 1. This assessment highlights the lack of reports on the *B. tequilensis*-mediated synthesis of ZnO nanoparticles.

**Table 1:** Comparative summary of *Bacillus-*mediated ZnO nanoparticle synthesis highlighting biological source, nanoparticle characteristics, and application areas.

*Bacillus* species	Biological source	Synthesis time (h)	NP morphology/size	Stability (Zeta potential)	Key characterization	Primary application	Reference
*Bacillus licheniformis* TT14s	Bacterial biomass	72	Hexagonal, 19.37 ± 5.28 nm	±2.5 mV	UV–Vis, FTIR, XRD, SEM	Cytotoxicity (glioblastoma cells)	^27^
*Bacillus paramycoides*	Cell-free supernatant	48	Hexagonal ZnO, ∼4–20 nm	−4.41 mV	UV–Vis, FTIR, XRD, TEM, DLS	Biomedical (antioxidant, antimicrobial, anticancer; in vivo)	^19^
*Bacillus safensis*	Cell-free filtrate	48	Spherical, ∼18 nm	-	UV–Vis, FTIR, XRD, SEM	Post-harvest disease control (*A. solani*, in apricot)	^28^
*Bacillus subtilis*	Cell-free supernatant	72	Irregular, 22–59 nm	−19.0 ± 4.3 mV	UV–Vis, FTIR, XRD, SEM	Antibacterial (foodborne pathogens)	^29^
*Bacillus cereus*	Cell free supernatant	Heated for 10 min	Spherical 21-35	29.5 ± 0.7,	UV–Vis, FTIR, Zeta potential, TEM, EDS	Antibacterial activity	^30^
				30.5 ± 1.1,			
				31.5 ± 0.9			
*Bacillus cereus*	Cell free supernatant	48	Irregular 58-63	−7.39 mV	UV–Vis, FTIR, Zeta potential, Zeta sizer, SEM	Antibacterial activity	^31^
*Bacillus haynesii*	Cell-free extract	24	Spherical, 50 ± 5 nm	-	UV–Vis, FTIR, XRD, SEM	Antimicrobial	^32^
*Bacillus megaterium* (NCIM 2326)	Cell-free supernatant	48	Anisotropic ZnO (size defined)	-	UV–Vis, FTIR, XRD, FESEM	Biomedical (nano-antibiotic; biosafety)	^33^
*Bacillus tequilensis*	Cell-free supernatant	48	Aggregates	−25 mV	UV–Vis, FTIR, XRD, Zeta potential, DLS, FESEM-EDS	Green synthesis and physiochemical characterization	Present study

## 2 Methods and methodology

### 2.1 Materials

Chemicals, including zinc sulphate monohydrate (ZnSO_4_·H_2_O M.W.:179.45 g/mol), Nutrient Agar, Nutrient Broth and Sodium Hydroxide (NaOH) pellets, were purchased from HiMedia Pvt. Ltd., India. Hydrochloric Acid was acquired from Loba Chemie Pvt. Ltd., India.

### 2.2 Isolation and identification of beneficial organism for microbial mediated synthesis of nanoparticles

The rhizosphere soil of *Tectona grandis* (Teak) trees was collected from agricultural bund plantations. The samples were serially diluted in Nutrient Agar plates, and the bacterial colonies were isolated and purified using the pure culture technique. Molecular identification was performed by 16 S rRNA sequencing and phylogenetic analysis at Medauxin Labs Pvt. Ltd., Bangalore, India. Genomic DNA was isolated and quantified using NanoDrop Spectrophotometer, and the quality was determined using a 0.8% agarose gel. PCR amplification of the 16 S rRNA gene was achieved using universal primers 27F (5′- AGAGTTTGATCCTGGCTCAG-3′) and 1492R (5′-TACGGYTACCTTGTTACGACTT-3′) was performed. Forward and reverse DNA sequencing reactions of PCR amplicon were carried out with forward and reverse primers using BDT v3.1 Cycle sequencing kit on ABI 3730xl Genetic Analyzer. The 16 S rRNA gene sequence was used to search the NCBI-Basic Local Alignment Search Tool database. Based on the maximum identity score, the first ten sequences were selected and aligned using the multiple alignment software program ClustalW. A distance matrix was generated, and a phylogenetic tree was constructed using MEGAXI.

### *2.3* *B. tequilensis* culture preparation for ZnO nanoparticle synthesis

The supernatant utilized for the synthesis of ZnO nanoparticles was prepared by growing bacterial culture in fresh nutrient broth (NB) containing (g/L) peptone 5 g, sodium chloride 5 g, HM peptone B (Equivalent to Beef extract) 1.5 g and yeast extract 1.5 g. A loopful of bacterial culture was inoculated into NB and incubated for four days. The culture was then centrifuged at 10,000 rpm for 10 min. The supernatant (cell-free filtrate) was collected in a conical flask.

### 2.4 Synthesis of ZnO nanoparticles

Extracellular synthesis of ZnO nanoparticles was carried out using *B. tequilensis* cell-free supernatant. The supernatant was mixed with zinc sulphate monohydrate (ZnSO_4_·H_2_O) solution at a ratio of 2:1, respectively, and incubated at room temperature for 48 h under static and dark conditions. The pH of the reaction mixture was maintained using 0.1 M NaOH. In the course of incubation, the appearance of white precipitate was considered a preliminary qualitative indication of ZnO nanoparticle formation. The precipitate was harvested by filtering through Whatman filter paper No. 1 and subsequently washed with sterile distilled water and ethanol. The filtrate was dried overnight at 60 °C in a hot air oven. The dried powder was acquired and further used for optimization and characterization.

In the current study, ZnO synthesis transpires under an alkaline condition using NaOH and ZnSO_4_ as the precursor. Nevertheless, the addition of bacterial supernatant provides a biologically active environment that stimulates nanoparticle nucleation, growth, and surface chemistry.

### 2.5 Optimization of synthesis parameters

The optimization of the synthesis parameters was based on the method described by Iqtedar et al.,^31^ using UV–Vis spectrophotometric analysis as the primary process to evaluate the nanoparticle synthesis.

The concentration of the precursor chemical was optimized by mixing the bacterial supernatant with ZnSO_4_·H_2_O solution in a 2:1 (v/v) ratio, resulting in final precursor concentrations of 5, 10, 15 and 20 mM. The reaction mixtures were incubated for 48 h under dark conditions.

For pH optimization, the reaction mixture was adjusted to pH 6, 8, and 10 using 1 M HCl and 1 M NaOH, while keeping all other reaction conditions constant.

To study the effect of temperature on the synthesis of nanoparticles, the reaction mixture was incubated at 30, 40, and 50 °C.

The optimal precursor concentration, pH and temperature were assessed by UV-Vis Spectroscopy analysis (300–800 nm) by comparing the sharpness and intensity of characteristic ZnO absorption peaks in the range of 320–360 nm, as mentioned in earlier studies.^34^

The yield percentage of ZnO nanoparticles was calculated by comparing the experimentally obtained dry mass with the theoretical yield based on the stoichiometric conversion of zinc sulphate monohydrate to ZnO. The yield percentage was determined using the following equation.^35^

$$Yield\;\left(\%\right)=\frac{Experimental\;weight\;of\;ZnO\;\left(mg\right)}{Theoritical\;weight\;of\;ZnO\;\left(mg\right)}\times 100$$

The theoretical yield of ZnO was calculated by assuming a 1:1 M conversion of Zn^2+^ ions to ZnO based on the initial precursor concentration.

### 2.6 Characterization of synthesized nanoparticles

The initial confirmation of *B. tequilensis*-assisted synthesis of ZnO nanoparticles was done by UV-Vis spectroscopy (LABMAN, LMSP-UV1200) by recording absorption spectra in the 300–800 nm wavelength range, as reported in earlier studies.^34^

Further characterization methodologies such as FTIR, Zeta potential analysis, FESEM-EDS, and XRD were performed at outsourced analytical facilities at Bharat Ratna Prof. C.N.R. Rao Research Centre, Avinashilingam Institute for Home Science and Higher Education for Women, Coimbatore, Tamil Nadu, India.

FTIR analysis was performed to identify the surface functional groups and confirm the formation of ZnO nanoparticles. The model used was SHIMADZU Miracle 10. The spectrum was recorded in the range of 4000–400 cm^−1^ using dried ZnO nanoparticle powder.

Dynamic Light Scattering (DLS) analysis was done to determine the size distribution of the nanoparticles. The ZnO nanoparticles were dispersed in ethanol and analyzed using the HORIBA Scientific SZ-100 nanoparticle analyzer. The stability of the nanoparticles in the colloidal suspension and their interactions with other charged particles were analyzed using the Zeta potential.^36^^,^^37^ The ZnO nanoparticles were dispersed in distilled water, and the suspension was diluted to an appropriate concentration by the instrument operator. The sample was sonicated for 10 min using a bath sonicator and analyzed at room temperature using the HORIBA Scientific SZ-100 nanoparticle analyzer.

To study the surface morphology, particle size distribution, and elemental composition of the synthesized ZnO nanoparticles, FESEM with EDS (TESCAN-MIRA3 XMU) was used. Images were acquired at different magnifications, and scale bars were provided in the corresponding micrographs.

The crystalline nature and approximate crystallite diameter of the ZnO nanoparticles were analyzed using XRD (X'Pert Pro PANalytical) with Cu Kα radiation (λ = 1.54 Å). The average crystallite size was calculated by Scherrer equation. The instrumental broadening was not subtracted; therefore, the crystallite values represent approximate estimates of the actual values.

### 2.7 Statistical analysis

The UV-Vis absorbance values were recorded in triplicate for each synthesis condition. Absorbance values at the characteristic wavelength (320 nm) were expressed as mean ± SD. Statistical differences in the optimization experiments were evaluated using one-way ANOVA performed in Microsoft Excel (Analysis ToolPak) at a significance level of p < 0.05.

## 3 Results and discussion

### 3.1 Isolation and identification of beneficial organism for microbial mediated synthesis of nanoparticles

**Fig. 1:**
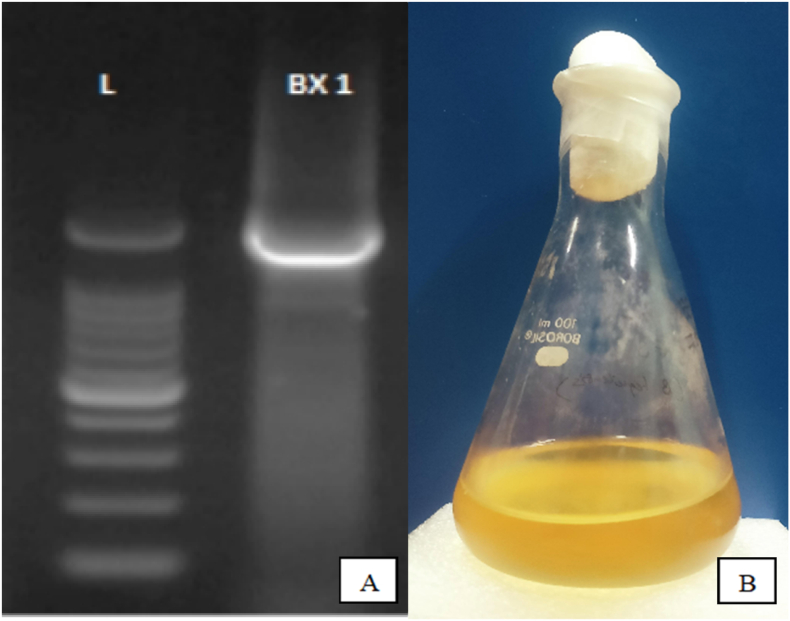
(A) Agarose (1.5%) gel electrophoresis of PCR using 27F and 1492R primers for bacterial samples. Legends: lad–Molecular Weight Marker–100 bp. (B) Broth culture of *Bacillus tequilensis* (BX1).

**Fig. 2:**
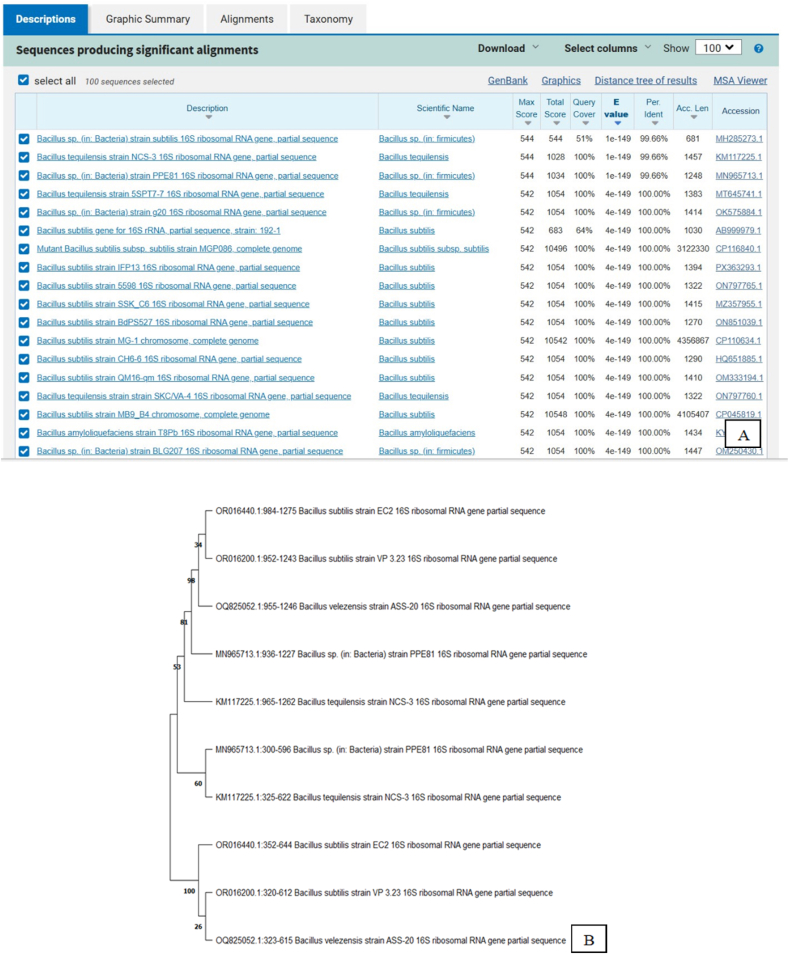
(A) Alignment view from NCBI GenBank of sample *Bacillus tequilensis(*BX1). (B) *Evolutionary relationships of Bacillustequilensis(*BX1) *with other taxa* using MEGAXI.

*Bacillus tequilensis* (BX1) was isolated from Teak (*Tectona grandis*) rhizosphere soil samples using serial dilution and pure culture techniques. PCR amplification of the 16 S rRNA gene with universal primers 27F and 1492R resulted in a single discrete PCR amplicon band of ∼1500 base pairs in agarose gel analysis (Fig. 1), verifying successful amplification. The obtained 16 S rRNA gene sequence was compared against the NCBI BLAST database (Fig. 2)

**Fig. 3:**
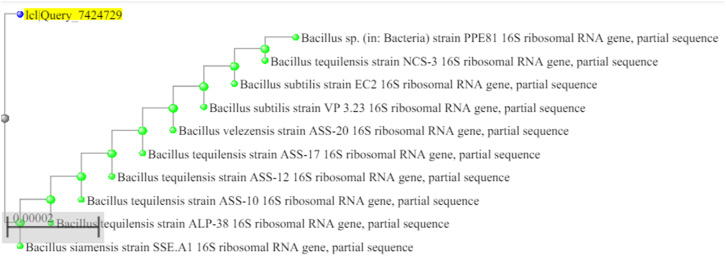
Distance tree view NCBI of sample *Bacillus tequilensis(*BX1).

Table 2 summarizes the BLAST results, including isolate codes, host plants, closest matching species, percentage identity, query coverage, and the corresponding NCBI accession numbers.

**Table 2:** BLAST-Based molecular identification of isolate *(*BX1) with NCBI Accession numbers.

Isolate code	Host Plant	Closest Matching Species	% Identity	Query Coverage	NCBI Accession No.
BX1	*Tectona grandis*	*Bacillus tequilensis*	99.65 %	100 %	PP851087

Molecular identification confirmed the taxonomic identity of the bacterial isolate, which was used for subsequent extracellular nanoparticle synthesis (see Fig. 3).

### 3.2 Synthesis of ZnO nanoparticles and optimization of synthesis parameters

#### 3.2.1 Effect of precursor concentration

**Fig. 4:**
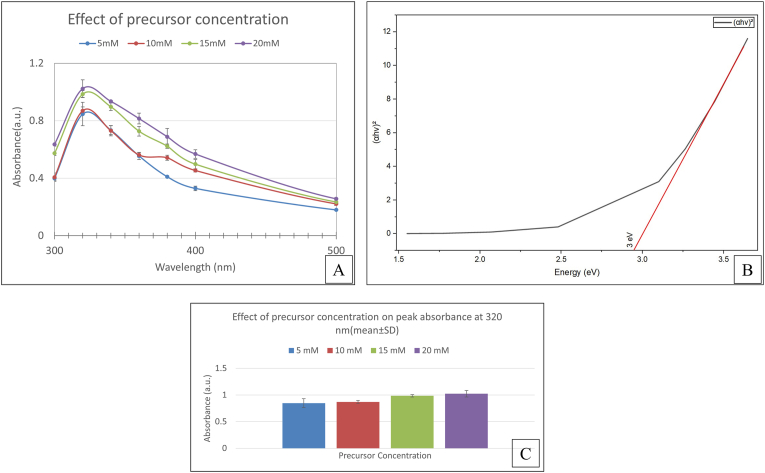
(A) UV–Vis absorption spectra of ZnO nanoparticles synthesized using different precursor concentrations (5-20 mM). Data points represent mean absorbance values ± SD (n = 3). (B) Tauc plot (αhν)^2^ versus hν for ZnO nanoparticles synthesized under optimized precursor concentration condition. (C) Bar graph showing mean absorbance ± SD at 320 nm for different precursor concentrations.

Different concentrations of ZnSO_4_·H_2_O resulted in different levels of ZnO-NP formation, as supported by the UV-Vis absorbance profiles. All precursor concentrations produced characteristic ZnO absorbance peaks in the range of 320-360 nm. The UV-Vis absorbance values at the characteristic ZnO wavelength (320 nm) were subjected to statistical analysis. One-way ANOVA revealed a statistically significant effect of precursor concentration on absorbance intensity (F = 7.65, p < 0.001). The mean absorbance increased with increasing precursor concentration, with 20 mM exhibiting the highest absorbance, suggesting efficient nanoparticle formation under this condition. The optical band gap of the ZnO nanoparticles was estimated using Tauc plot analysis (Origin Software 2026) by extrapolating of the linear region of the (αhν)^2^ versus hν plot,^38^ yielding a band gap energy of approximately 3.0 eV (Fig. 4). As the film thickness was not precisely controlled, the estimated band-gap values should be considered approximate.

**Fig. 5:**
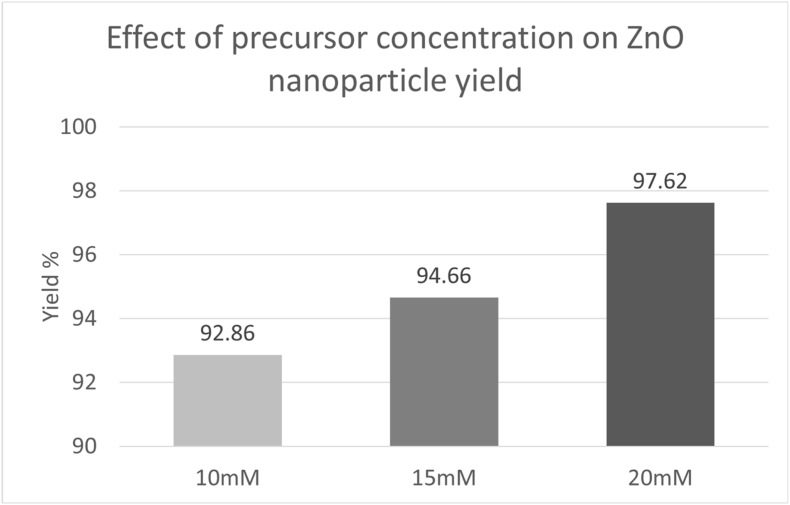
Effect of precursor concentration on ZnO nanoparticle yield.

The yield % of ZnO nanoparticles varied with the precursor concentration (Fig. 5). Among the tested concentrations, the highest yield was obtained at 20 mM concentration. The yield trend was consistent with the UV-Vis absorbance results.

Based on the spectroscopic, statistical, and yield % estimation, a precursor concentration of 20 mM was selected as the optimal condition for ZnO nanoparticle synthesis. At this concentration, UV-Vis analysis displayed the highest absorbance intensity at 320 nm, which was statistically significant, as confirmed by one-way ANOVA. Yield percentage analysis supported this selection by demonstrating the highest conversion efficiency of the selected factor.

#### 3.2.2 Effect of pH

To optimize the pH of the reaction mixture, the reaction was carried out using a previously optimized 20 mM zinc sulphate concentration under varying pH conditions.

**Fig. 6:**
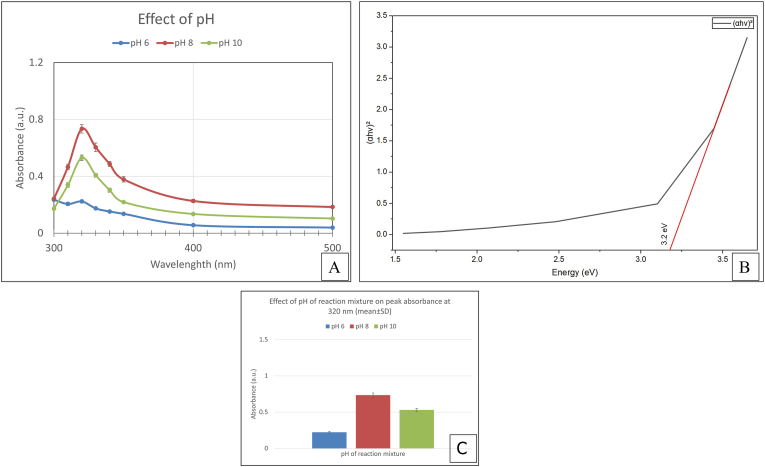
(A) UV–Vis absorption spectra of ZnO nanoparticle synthesized using different pH of reaction mixture (pH 6,8, 10). Data points represent mean absorbance values ± SD (n = 3). (B) Tauc plot (αhν)^2^ versus hν for ZnO nanoparticles synthesized under optimized pH of reaction mixture (C) Bar graph showing mean absorbance ± SD at 320 nm for different pH values of the reaction mixture.

The UV-Vis absorbance values at the characteristic ZnO wavelength (320 nm) were subjected to statistical analysis. One-way ANOVA exhibited a statistically significant effect of precursor concentration on absorbance intensity (F = 429.9, p < 0.001). The maximum absorbance was observed at pH 8, suggesting that alkaline conditions favoured nanoparticle formation. The optical band gap of the ZnO nanoparticles was calculated using Tauc plot analysis using Origin Software 2026. The extrapolation of the linear region of the (αhν)^2^ versus hν plot^38^ yielded a band gap energy of approximately 3.2 eV (Fig. 6). As the film thickness was not precisely controlled, the estimated band gap values should be considered approximate.

**Fig. 7:**
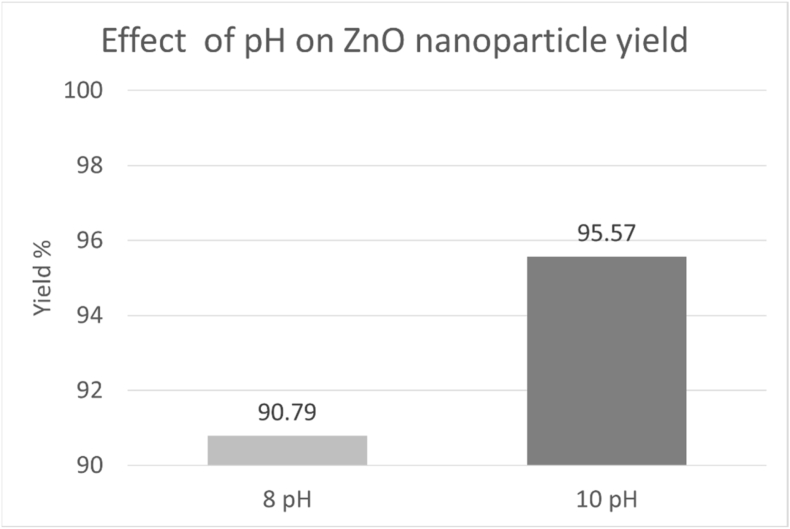
Effect of pH on ZnO nanoparticle yield.

The yield % of ZnO nanoparticles varied with the pH of the reaction mixture (Fig. 7). Among the tested concentrations, the highest yield was obtained at pH 10. The yield trend was consistent with the UV-Vis absorbance results.

Based on the collective evaluation of spectroscopic and statistical analyses, a pH 8 of the reaction mixture was selected as the optimal condition for ZnO nanoparticle synthesis. At this concentration, UV-Vis analysis exhibited the highest absorbance intensity at 320 nm, which was statistically significant, as confirmed by one-way ANOVA. The increased yield at pH 10 is possibly linked to enhanced hydroxide-facilitated precipitation and aggregation, which may contribute to a higher yield but reduced control over nanoparticle characteristics. Thus, pH 8 was selected as the optimal condition for subsequent synthesis and characterization based on optical properties and nanoparticle quality, rather than yield alone.

#### 3.2.3 Effect of temperature

**Fig. 8:**
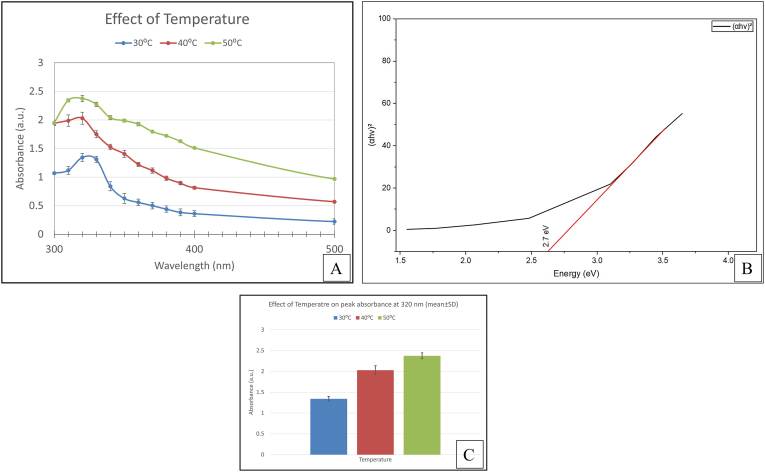
(A) UV Vis absorption spectra of ZnO nanoparticle synthesized using different temperature of reaction mixture (30 °C, 40 °C, 50 °C). Data points represent mean absorbance values ± SD (n = 3). (B) Tauc plot (αhν)^2^ versus hν for ZnO nanoparticle synthesized under optimized temperature of reaction mixture. (C) Bar graph showing mean absorbance ± SD at 320 nm for different temperature of reaction mixture.

The effect of incubation temperature on nanoparticle synthesis was evaluated by testing varying temperatures namely, 30, 40, and 50 °C. Visual inspection revealed nanoparticle synthesis at all temperatures. The UV-Vis analysis showed that all temperatures exhibited characteristic absorbance peaks. Among them, an incubation temperature of 50 °C was chosen as the optimal temperature, as it showed a sharp peak at 320 nm. UV-Vis absorbance values at the characteristic ZnO wavelength were subjected to statistical analysis. One-way ANOVA showed a statistically significant effect of precursor concentration on absorbance intensity (F = 129.8, p < 0.001). The maximum absorbance was observed at 50 °C. The optical band gap of the ZnO nanoparticles was calculated using Tauc plot analysis using Origin Software 2026. The extrapolation of the linear region of the (αhν)^2^ versus hν plot^38^ yielded a band gap energy of approximately 2.7 eV (Fig. 8). The estimated band gap should be viewed as approximate, as the film thickness was not precisely controlled.

**Fig. 9:**
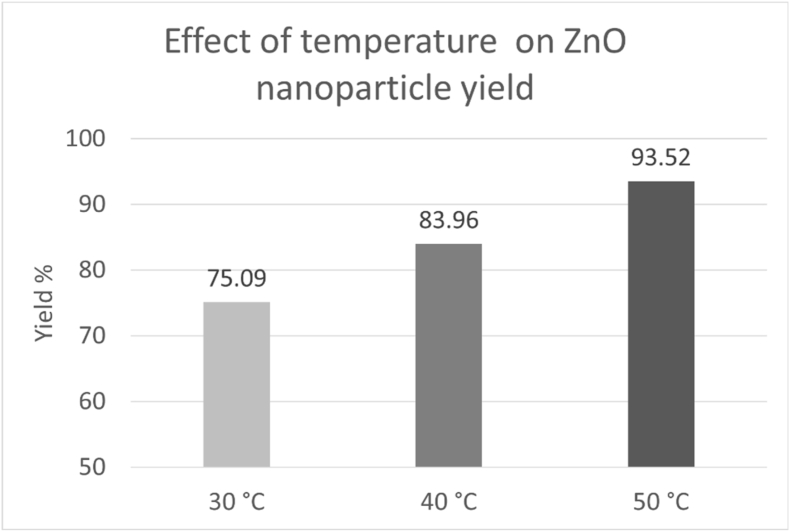
Effect of temperature on ZnO nanoparticle yield.

The yield % of ZnO nanoparticles showed variation with the temperature of the reaction mixture (Fig. 9). Among the tested concentrations, the highest yield was obtained for 50 °C. The yield trend was consistent with UV-Vis absorbance results.

Based on the collective evaluation of spectroscopic, statistical and yield % estimation, the temperature of 50 °C of reaction mixture was selected as the optimal condition for ZnO nanoparticle synthesis. At this concentration, UV-Vis analysis showed the highest absorbance intensity at 320 nm, which was statistically significant as confirmed by one-way ANOVA. Yield percentage analysis supported this selection by demonstrating the highest conversion efficiency of the selected factor. Therefore, 50 °C was selected as the optimized temperature.

The summary of optimized synthesis parameters for bacterial supernatant-assisted ZNO nanoparticle synthesis is shown in Table 3.

**Table 3:** Optimized synthesis parameters for ZnO nanoparticle formation and selection criteria.

Synthesis parameter	Values	Optimal value selected	Experimental justification
Precursor concentration	5, 10, 15, 20 mM	20 mM	Produced a well-defined UV Vis absorbance peak at 320 nm and better yield when compared with other concentrations.
pH of reaction mixture	pH 6, 8, 10	pH 8	Mildly alkaline pH promoted sharper UV Vis absorbance peak.
Temperature of reaction mixture	30 °C, 40 °C, 50 °C	50 °C	Elevated temperature produced sharper UV Vis absorbance peak and increased yield when compared to other temperatures.

**Table 4:** FTIR functional groups analysis of bacterial supernatant and synthesized ZnO nanoparticles.

Spectra	Functional Groups present in supernatant	Functional groups present in ZnO nanoparticle
3300–3400 cm^−1^	O–H Stretching vibrations of hydroxyl groups	O–H Stretching vibrations of hydroxyl groups
1630–1650 cm^−1^	C=O stretching vibration of proteins (Amide I band)	C=O stretching vibration of proteins (Amide I band)
1388 cm^−1^	-	C–H bending vibrations
1045 cm^−1^	C–O stretching vibration	-
400 and 600 cm^−1^	-	ZnO stretching vibrations

According to the UV-Vis spectral analysis, the synthesis parameters were systematically optimized to attain reproducible ZnO nanoparticle formation (see Table 4). The optimal conditions were determined to be a precursor concentration of 20 mM, pH 8, and a reaction temperature of 50 °C, as these conditions consistently produced a characteristic ZnO absorption peak in the range of 320-360 nm. Therefore, these optimized parameters were employed for all successive nanoparticle synthesis and characterization studies. One-way ANOVA established that the precursor concentration, pH, and temperature had a statistically significant effect on the absorbance intensity. Disparities in the yield percentage were observed under different conditions; balanced parameters were selected for reproducibility and quality. Consequently, the optimized conditions were employed for the successive nanoparticle and characterization.

#### 3.2.4 FTIR

FTIR analysis was performed to compare the functional groups present in the bacterial supernatant (ES B) and *B. tequilensis*-assisted ZnO nanoparticles (ES 4) to assess the functional groups associated with biogenic synthesis.

##### 3.2.4.1 FTIR of bacterial supernatant (ES B)

**Fig. 10:**
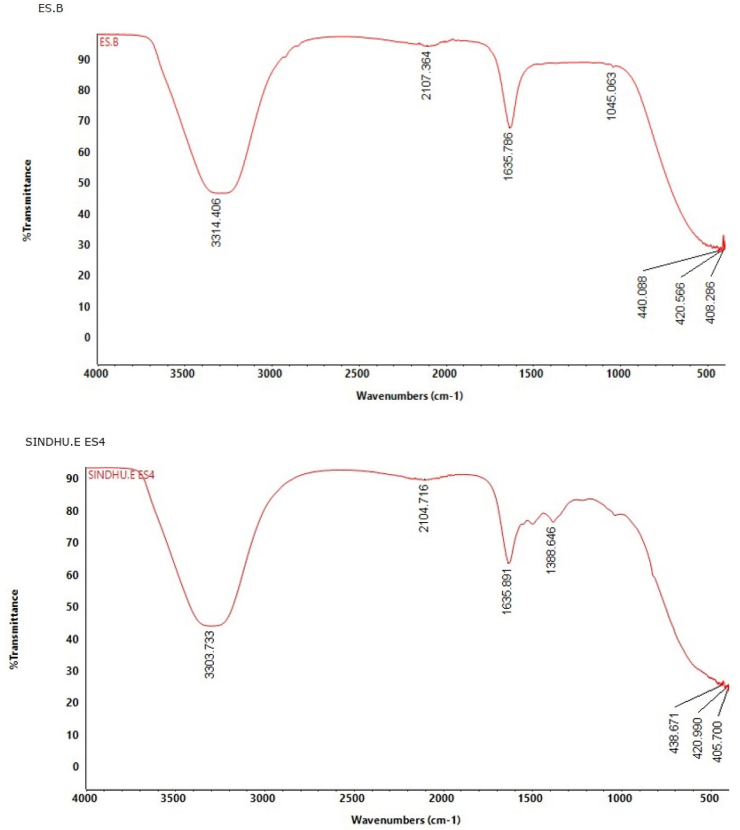
FTIR spectra of bacterial supernatant (ES B) and synthesized ZnO nanoparticles (ES 4).

The FTIR spectrum of the bacterial supernatant (Fig. 10) showed a broad absorption band at 3300 cm^−1^, corresponding to O–H and N–H stretching vibrations, suggestive of the presence of hydroxyl and amine groups.^39^^,^^40^ A band around 1635 cm^−1^ can be ascribed to the C=O stretching vibrations of amide groups,^41^^,^^42^ while bands around 1000–1100 cm^−1^ region may be associated with C–O stretching vibrations^43^, ^44^, ^45^, ^46^, indicating the multifaceted organic composition of the supernatant. Low-wavelength bands in the 400–450 cm^−1^ region were also detected, which may arise from background inorganic constituents commonly present in complex biological media.

##### 3.2.4.2 FTIR of synthesized ZnO nanoparticles (ES 4)

The FTIR spectrum of *B. tequilensis*-assisted ZnO nanoparticles (Fig. 10) showed an absorption band analogous to those observed in the bacterial supernatant, including bands at 3300 cm^−1^ and 1635 cm^−1^, demonstrating the persistence of organic functional groups post-synthesis. The amide group suggests the involvement of proteins in the stabilization of nanoparticles.^41^^,^^46^ In particularly, the band at 1045 cm^−1^ detected in the supernatant was absent in the ZnO nanoparticle spectrum, while a new absorption band appeared at 1388 cm^−1^, equivalent to C–H bending vibrations.^47^^,^^48^ Peaks observed between 400 and 600 cm^−1^ are attributed to metal-oxygen bond vibrations^49^^,^^50^, confirming the synthesis of ZnO nanoparticles with the assistance of *B*. *tequilensis*.

Comparative FTIR analysis revealed that the bacterial supernatant contains biomolecules that may contribute to the surface functionalization of the synthesized ZnO nanoparticles. These surface-affiliated functional groups may influence nanoparticle growth and stabilization through absorption or capping effects, as observed in the biogenic synthesis pathway. Therefore, FTIR analysis, offers a qualitative understanding of the surface chemistry of biogenic ZnO nanoparticles.

###### 3.2.4.2.1 Proposed mechanism of bacterial supernatant-mediated ZnO nanoparticle synthesis

**Fig. 11:**
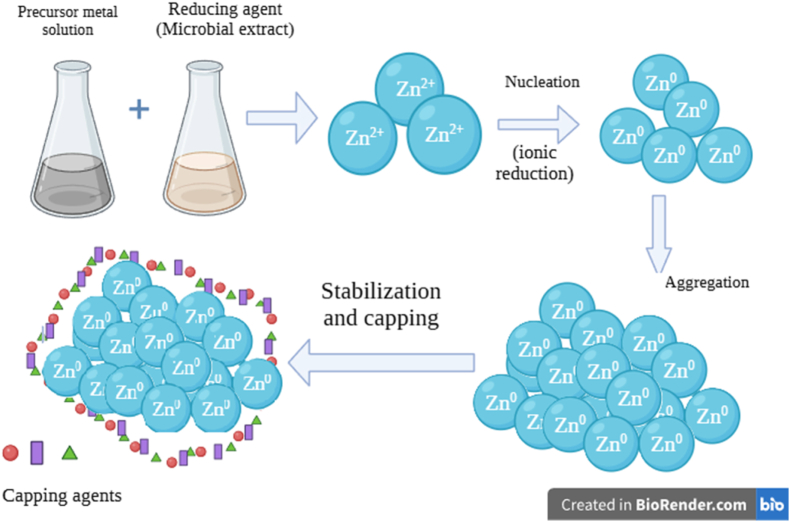
Proposed mechanism of bacterial supernatant-mediated ZnO nanoparticle synthesis.

The mechanism of biogenic synthesis of nanoparticles remains under active investigation owing to the complex nature of biological extracts.^51^ Fig. 11 presents a schematic representation of the proposed extracellular biosynthesis mechanism of the nanoparticles.

Microorganisms possess an innate capability to synthesize nanoparticles through either intra- or extracellular pathways.^52^ In the present study, ZnO synthesis followed an extracellular pathway, wherein biomolecules like proteins present in the bacterial supernatant are proposed to assist in the reduction and capping of nanoparticles.^51^ FTIR demonstrated the occurrence of functional groups, including hydroxyl and amide groups, associated with these biomolecules.

There are three stages in the green synthesis of nanoparticles: nucleation, growth and stabilization. In the nucleation phase, Zn^2+^ ions dissociate from the precursor solution by interacting with the supernatant.^53^ Under aqueous and alkaline conditions, Zn^2+^ ions undergo hydrolysis to form zinc aqua or zinc hydroxo complexes, which are subsequently converted to zinc hydroxide intermediates [Zn (OH)_2_], acting as precursors for ZnO formation.^54^ The alcohol (OH) groups present on the cell surface are involved in signalling pathways to the precursor for their reduction.^55^ After the reduction of Zn ^2+^ to zero valent zinc atoms (Zn^0^), the zinc complex is further converted into ZnO nanoparticles during annealing.^52^ In the growth phase, the metal oxide nanoparticles aggregate by interacting with biomolecules in the supernatant. Finally, the extracellular biomolecules released by bacteria probably cap and stabilize the nanoparticles.^53^^,^^56^ The proposed mechanism underlines a biomolecule-aided pathway, wherein the bacterial supernatant modulates the formation of ZnO nanoparticles through surface functionalization. Although hydroxide-mediated conversion (chemical synthesis) of zinc precursors contributes to ZnO formation, extracellular biomolecules are proposed to regulate nucleation, aggregation, and surface properties, resulting in the physical and chemical characteristics observed in the synthesized ZnO nanoparticles.

The present study focuses on understanding the effect of bacterial supernatant on ZnO nanoparticle growth, aggregation and surface characteristics, rather than distinguishing ZnO formation from a purely chemical precipitation pathway. The proposed mechanism emphasizes the role of bacterial supernatant in facilitating ZnO nanoparticle synthesis, which is in agreement with the FTIR analysis and previous reports on the extracellular synthesis of ZnO nanoparticles.

#### 3.2.5 DLS analysis

**Fig. 12:**
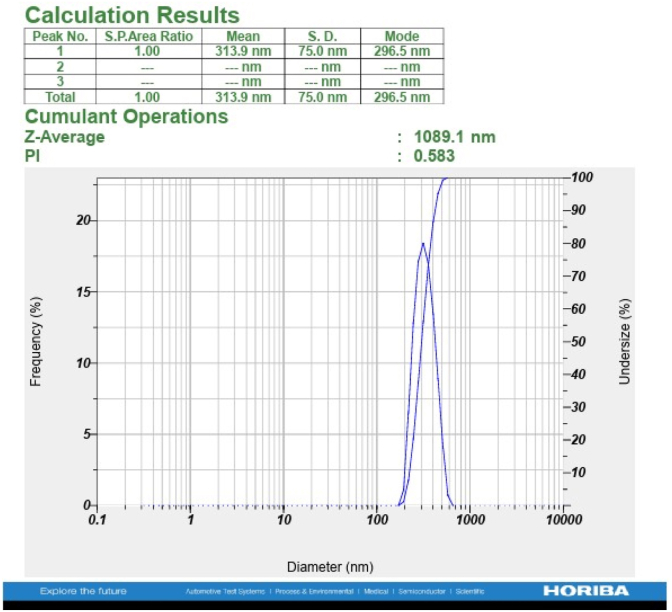
DLS-derived hydrodynamic size distribution of *Bacillus tequilensis* supernatant-assisted ZnO nanoparticles.

The ZnO nanoparticles were dispersed in ethanol and their particle size distribution evaluated. DLS analysis of the ZnO nanoparticles showed a mean hydrodynamic diameter of approximately 313 nm and a Z-average diameter of 1089 nm (Fig. 12). The polydispersity index (PDI) of the nanoparticle suspension was 0.583, indicating a polydisperse system with significant aggregation in dispersion. The high Z-average diameter is probably due to the effect of larger aggregates, as DLS measurements are weighted towards particles with higher scattering intensities.

FSESEM analysis also demonstrated irregular and agglomerated ZnO nanoparticles, while XRD analysis exhibited an average crystallite size of approximately 18.64 nm. The variation in particle size acquired from DLS, FESEM and XRD is due to the different measurement principles of these techniques.

These results collectively establish that the synthesized ZnO nanoparticles consist of crystallites assembled into larger agglomerates, consistent with the behaviour commonly observed in biogenic nanoparticles.

#### 3.2.6 Zeta analysis

For the zeta potential analysis, the ZnO nanoparticle powder was dispersed in distilled water to obtain a colloidal suspension. The suspension was sonicated using a bath sonicator for 30 min to ensure uniform dispersion. Zeta potential was measured using the HORIBA Scientific SZ-100 nanoparticle analyzer at an outsourced analytical laboratory.

**Fig. 13:**
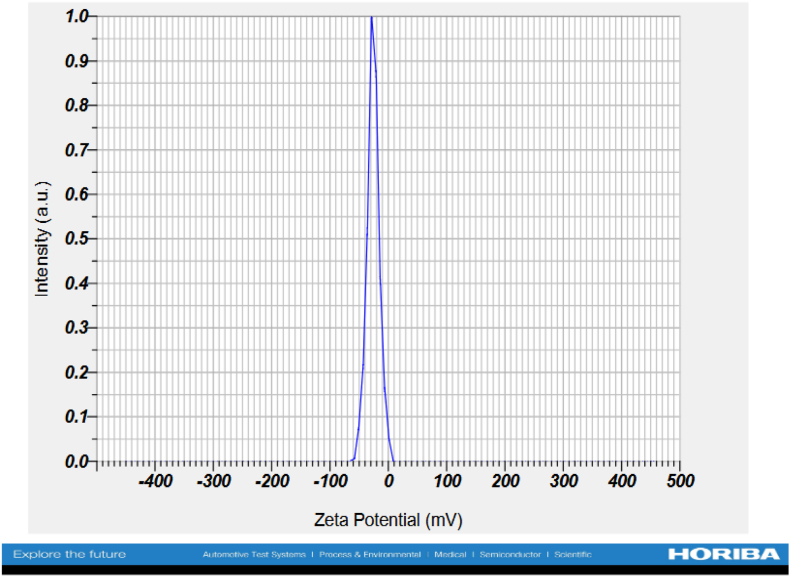
Zeta potential of the synthesized ZnO nanoparticles.

The ZnO nanoparticles exhibited a zeta potential of −25.8 mV (Fig. 13), indicating moderate stability. Similarly, a study^57^ reported a zeta potential of −25mV for ZnO nanoparticles synthesized using *Moringa oleifera* seed extract. Studies have demonstrated that zeta potential values greater than +30 mV or lower than −30 mV stimulate strong electrostatic repulsion between particles, thus successfully preventing their agglomeration.^58^^,^^59^ The negative zeta potential also indicates that the nanoparticles possess considerable electrostatic forces.^60^ Other studies^61^, ^62^, ^63^ have shown that nanoparticles with −25 mV and +25 mV are likely to be more stable.

#### 3.2.7 FESEM-EDS

**Fig. 14:**
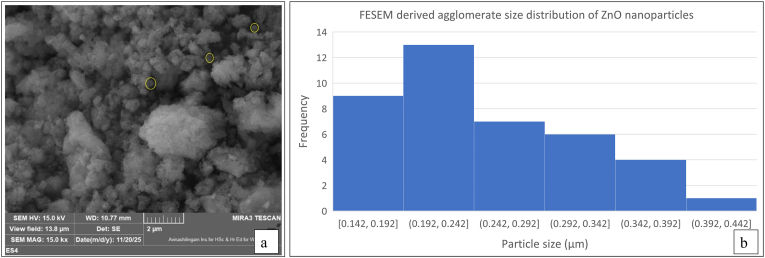
(a) FESEM micrograph of the synthesized ZnO nanoparticles (ES 4) showing an agglomerated morphology (magnification × 15,000; scale bar = 2 μm). Representative agglomerates selected for size measurements are highlighted. (b) Agglomerate size distribution histogram of ZnO nanoparticles measured from FESEM micrographs at a 2 μm scale.

Field-emission scanning electron microscopy (FESEM) was used to observe the surface morphology and size characteristics of the synthesized ZnO nanoparticles (ES 4). FESEM micrographs showed that the biosynthesized nanoparticles displayed irregular, largely agglomerated structures (Fig. 14). The individual primary particles were not noticeably separated. The particles were visible as predominantly clustered aggregates, which is commonly reported for biologically synthesized nanoparticles^64^, ^65^, ^66^, ^67^, ^68^, as they possess a higher surface area and durable affinity among them.^69^ This observed agglomeration can be attributed to the polarity and electrostatic attraction of ZnO nanoparticles, which causes the particles to stick together.^48^^,^^49^ Furthermore, drying and solvent evaporation during the sample preparation for FESEM analysis may promote particle clustering.

The measurement of agglomerates was carried out directly from FESEM images acquired at a 2 μm scale, and the corresponding agglomerated size distribution histogram was generated using ImageJ software (Fig. 14). The histogram indicates that the aggregate sizes mainly ranged between approximately 0.14 and 0.44 μm, confirming the formation of polydisperse nanoparticle clusters instead of segregated primary particles.

**Fig. 15:**
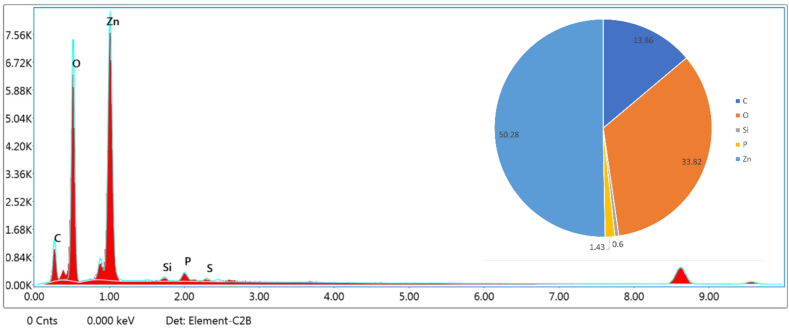
EDS spectrum and elemental composition of synthesized ZnO nanoparticles.

The elemental structure of the synthesized ZnO nanoparticles (ES 4) was investigated using EDS analysis. The EDS spectra revealed that the synthesized ZnO nanoparticles contained Zn and O as major elements, with weight percentages of 50.28% and 33.82%, respectively, validating the successful synthesis of ZnO. Minor signals analogous to C, Si, and P with weight percentages of 13.86%, 0.60%, and 1.43%, respectively, were also detected (Fig. 15). The C signal can be attributed to multiple sources that are commonly reported in nanoparticle synthesis. Carbon input may arise from residual organic molecules associated with the biological synthesis medium, as biogenic ZnO nanoparticles are often capped by compounds containing C–H and C–O bonds.^70^^,^^71^ In addition, weak carbon signals can originate from experimental and instrumental factors, such as the use of carbon tape for mounting the sample for EDS analysis^72^ or carbon coatings and integral carbon contributions related to the EDS detector and sample holder.^73^ The Si signal may originate from the Si grids used for loading the samples.^74^ The trace presence of P may be attributed to residual phosphate-containing biomolecules or metabolites associated with microbial synthesis. Similar minor P signals have been reported in EDS analysis of biologically synthesized ZnO nanoparticles using plant extracts.^75^

The low concentrations of these elements indicate that they are present only as minor surface-related or background contributions and do not affect the phase purity of the synthesized ZnO nanoparticles.

#### 3.2.8 XRD analysis

**Fig. 16:**
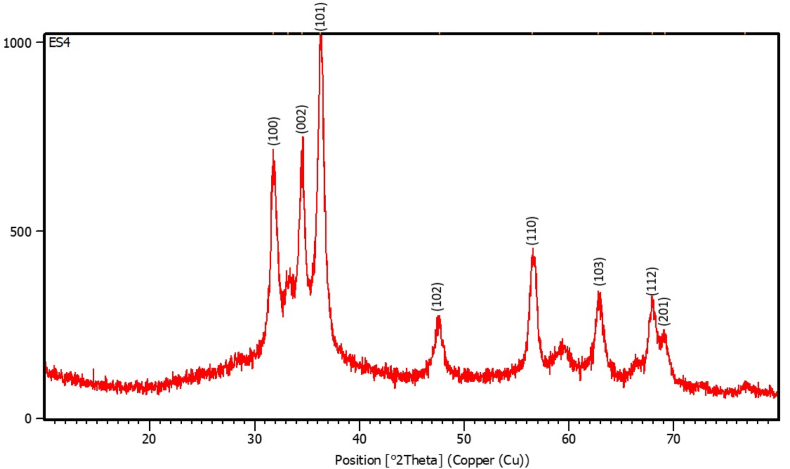
XRD pattern of ZnO nanoparticles showing characteristic diffraction peaks indexed to the hexagonal wurtzite ZnO structure with corresponding Miller indices (hkl).

The crystalline nature of the ZnO nanoparticles (ES 4) was examined using X-ray diffraction (XRD). The diffraction pattern displayed distinct peaks at 2θ values of 31.7**°**, 34.5**°**, 36.2**°**, 47.6**°**, 56.4**°**, 62.7**°**, 67.9**°**, and 76.8**°** (Table 5), which were indexed to the (100), (002), (101), (102), (110), (103), (112) and (201) Miller indices, respectively (Fig. 16). These peaks are in agreement with the standard hexagonal wurtzite structure of ZnO (JCPDS No. 36–1451), confirming the formation of crystalline ZnO nanoparticles.^76^, ^77^, ^78^ This observation is in accordance with previous reports on biologically synthesized ZnO nanoparticles.^1^^,^^40^ No additional impurity peaks corresponding to Zn (OH)_2_ or other zinc phases were observed, signifying the high phase purity of the synthesized ZnO nanoparticles. The identification of the ZnO phase in the present study was based on crystallographic evidence from XRD and characteristic Zn–O stretching vibrations observed in the FTIR spectrum, without the application of post-synthesis high-temperature calcination.

**Table 5:** XRD peak parameters of synthesized ZnO nanoparticles.

Pos. [°2Th.]	Height [cts]	FWHM Left [°2Th.]	d-spacing [Å]	Rel. Int. [%]	Size D (nm)
31.7704	476.33	0.6022	2.81662	59.84	13.72
33.2198	174.74	0.4015	2.69696	21.95	20.65
34.5005	495.83	0.4684	2.59972	62.29	17.76
36.2599	796.01	0.2342	2.47751	100.00	35.69
47.6850	131.77	0.4015	1.90720	16.55	21.63
56.4830	273.72	0.6022	1.62924	34.39	14.97
62.7989	187.42	0.6022	1.47972	23.54	15.46
67.9538	180.28	0.6022	1.37948	22.65	15.91
69.1256	99.76	0.5353	1.35894	12.53	18.02
76.8239	17.69	0.8029	1.24082	2.22	12.63

The average crystallite size was determined using Scherrer's equation.^79^

$$D=\frac{K\lambda }{\beta \;\cos\theta }$$

where D is the crystalline size, K is Scherrer's constant (0.9), λ is the wavelength of the X-ray source (1.54), β is the full width at half maximum (FWHM) of the diffraction peak, and θ is the Bragg angle.

Due to the unavailability of instrumental broadening data from the outsourced analysis, the crystallite size estimation is approximate. Based on the analysis, the average crystallite size of the ZnO nanoparticles was approximately 18.64 nm, as assessed using Scherrer's equation.

The discrepancy between the FESEM agglomerate sizes and XRD crystallite size arises from the aggregation of numerous crystallites into larger clusters. Such aggregation is commonly observed in the biologically assisted synthesis of ZnO nanoparticles because of surface energy effects and sample preparation conditions.

## 4 Conclusion

In the current study, ZnO nanoparticles were successfully synthesized using the bacterial supernatant of *Bacillus tequilensis*. To the best of our knowledge, this appears to be the first report demonstrating *B. tequilensis* supernatant-assisted synthesis and optimization of ZnO nanoparticles. The prefatory optimization of the synthesis parameters using UV-Vis spectroscopy identified 20 mM precursor concentration, pH 8, and temperature of 50 °C of the reaction mixture as optimal conditions, producing a characteristic absorption band in the 320−360 nm range. The synthesis parameters had a significant influence on nanoparticle formation, as demonstrated by one-way ANOVA. In addition, the yield % analysis verified the maximum nanoparticle recovery under the optimized conditions. The presence of hydroxyl, amide, and other functional groups, as indicated by FTIR analysis, suggests their role in the reduction and stabilization of nanoparticles. The synthesized nanoparticles had a zeta potential of −25.8 mV, indicating moderate colloidal stability. Consequently, aggregation in dispersion was also present, as indicated by the DLS analysis results, which revealed a mean hydrodynamic diameter, Z average, and PDI of approximately 313 nm, 1089 nm, and 0.583, respectively. Similarly, FESEM analysis revealed irregular and aggregated particles, while XRD analysis exhibited a crystalline hexagonal wurtzite structure with an average crystallite size of approximately 18 nm. The EDS study confirmed the presence of Zn and O as the major elements in the synthesized nanoparticles.

The findings of this study establish that *Bacillus tequilensis* supernatant can be successfully utilized to supplement the synthesis of ZnO nanoparticles by providing functional groups from extracellular biomolecules, which augments the reduction and stabilization process. Hence, it has the potential to be a sustainable alternative for green nanomaterial production.

## 5 Limitations and future prospects

The present study mainly focused on the biogenic-assisted synthesis, optimization, and physical and chemical characterization of ZnO nanoparticles using the cell-free supernatant of *Bacillus tequilensis*. One limitation of this study is that the quantitative estimation of individual extracellular biomolecules and real-time monitoring of reaction kinetics were not performed because of the complex and heterogeneous nature of bacterial metabolites. Another limitation is the absence of a control reaction conducted without bacterial supernatant; therefore, future investigations should include such controls to further validate the proposed biomolecule-mediated ZnO nanoparticle formation mechanism. Moreover, FESEM and DLS analyses indicated nanoparticle aggregation, which may affect dispersion behaviour; hence, strategies to improve colloidal stability and reduce aggregation necessitate further investigation. Furthermore, practical biological evaluations, including antibacterial, antioxidant, or cytotoxicity assays, were not included and remain beyond the scope of the current investigation. Although the synthesis process was optimized at the laboratory scale, the probable challenges associated with large-scale production and long-term storage stability were not addressed and require further investigation. Future research should emphasize biological validation, consider biomolecule-nanoparticle interactions, and evaluate application-oriented performance. By addressing these gaps and limitations, *Bacillus tequilensis*-assisted ZnO nanoparticle synthesis can be gradually rendered practical and scalable for nano-biotechnological applications.

## CRediT authorship contribution statement

**Elayappan Sindhu:** Writing – original draft, Visualization, Methodology, Investigation, Formal analysis, Data curation, Conceptualization. **Arumugam Karthikeyan:** Writing – review & editing, Validation, Supervision, Formal analysis.

## Funding

The research was supported by Junior Research Fellowship from University Grant Commission (UGC), Government of India (award number 210510879937).

## Declaration of competing interest

The authors declare that they have no known competing financial interests or personal relationships associated with this publication that could have appeared to influence the work reported in this paper. They also declare that this manuscript has not been published elsewhere.

## Data Availability

Data will be made available on request.
